# More Than Meets the Eye: Art Engages the Social Brain

**DOI:** 10.3389/fnins.2022.738865

**Published:** 2022-02-25

**Authors:** Janneke E. P. van Leeuwen, Jeroen Boomgaard, Danilo Bzdok, Sebastian J. Crutch, Jason D. Warren

**Affiliations:** ^1^Dementia Research Centre, UCL Queen Square Institute of Neurology, University College London, London, United Kingdom; ^2^The Thinking Eye, ACAVA Limehouse Arts Foundation, London, United Kingdom; ^3^Research Group Art and Public Space, Gerrit Rietveld Academie, Amsterdam, Netherlands; ^4^Department of Biomedical Engineering, McGill University, Montréal, ON, Canada

**Keywords:** art, neuroaesthetics, creativity, social brain, connectome, mental health, dementia, art therapy

## Abstract

Here we present the viewpoint that art essentially engages the social brain, by demonstrating how art processing maps onto the social brain connectome—the most comprehensive diagram of the neural dynamics that regulate human social cognition to date. We start with a brief history of the rise of neuroaesthetics as the scientific study of art perception and appreciation, in relation to developments in contemporary art practice and theory during the same period. Building further on a growing awareness of the importance of social context in art production and appreciation, we then set out how art engages the social brain and outline candidate components of the “artistic brain connectome.” We explain how our functional model for art as a social brain phenomenon may operate when engaging with artworks. We call for closer collaborations between the burgeoning field of neuroaesthetics and arts professionals, cultural institutions and diverse audiences in order to fully delineate and contextualize this model. Complementary to the unquestionable value of art for art’s sake, we argue that its neural grounding in the social brain raises important practical implications for mental health, and the care of people living with dementia and other neurological conditions.

## Introduction: Placing Art in the World and in the Brain

While beauty has been the subject of philosophical enquiry since ancient times (e.g., Aristotle’s Metaphysics and Plotinus’ Enneads), aesthetics as the study of “what is sensed and imagined” was founded by the German philosopher Alexander Gottlieb Baumgarten in 1735 (Baumgarten, Meditationes § CXVI, pp. 86–7). Its emergence coincided with the Enlightenment, during which rational thought was considered the only reliable method to uncover universal truths. Through seminal publications such as The Critique of Judgment by Kant (1790), the subject of aesthetics gradually became “the nature and appreciation of beauty.” According to Kant, beautiful art evoked universal pleasure, disconnected from personal interest. This contention was adopted as the guiding principle in the creation, as well as the cultural analysis, of the “fine arts” in Western societies throughout the eighteenth and nineteenth centuries. Over the course of the twentieth century, however, progressive artists led a movement away from this dogma of aesthetics. They recognized that art communicated on deeper and more complex levels with the human mind, in ways that went beyond the experience of beauty and pleasure. In her seminal performance “Art Must Be Beautiful, Artist Must Be Beautiful,” the artist Marina Abramović provocatively challenged the notion that art must be beautiful, proclaiming that this expectation applied to (female) artists as well (Abramović, 1975). Abramović’s pivotal performance revealed that the aesthetic judgment of art is not primarily conveyed by the senses or guided by universal attributes of beauty, but is inevitably influenced by subjective and ever-changing social norms specifying what art, and artists, “should” be like. Growing acknowledgment of this critical relationship between art and its social context has had a profound influence on subsequent artistic practice and academic study.

Though philosophers and scientists have long been interested in how we perceive and experience art, neuroaesthetics as a scientific discipline addressing the neural bases for the perception, contemplation and creation of art is a very recent development (Zeki and Nash, 1999; Cela-Conde et al., 2004; Kawabata and Zeki, 2004; Marin, 2015). Neuroaesthetics had its roots in visual neuroscience, which is reflected by codifications such as the “eight laws” of artistic experience proposed by Ramachandran and Hirstein (1999), though the reductionism of this approach has been eloquently criticized (Tallis, 2008). From a philosophical perspective, the research paradigms of neuroaesthestics are largely in the tradition of Kant, focusing on the beauty and pleasure or reward value of (visual) art. In the past decade, however, researchers have become increasingly aware of the need for broader conceptual frameworks that address a greater diversity of aesthetic objects, and which contextualize art beyond the neural mechanisms of sensori-motor processing and the experience of beauty and pleasure (Marin, 2015; Pearce et al., 2016). Pearce et al. (2016) have proposed that the cognitive neuroscience of aesthetics, the cognitive neuroscience of art and the cognitive neuroscience of beauty constitute overlapping, but distinct subfields of neuroaesthetics (Pearce et al., 2016). Iigaya et al. (2020) have suggested that computational methods may further elucidate feature-based neural mechanisms of art perception and appreciation, but that closer collaborations with researchers and practitioners in the arts and related humanities will be needed to attain a comprehensive understanding of the richness and complexity of art. Acknowledging that we are not passive perceivers of art, but engage with it dynamically as a social artifact, may be especially important for elucidating the neural mechanisms of artistic creativity. Neuroaesthetics has been criticized for not taking the social context and value of art enough into account (Sherman and Morrissey, 2017), but Skov and Nadal (2017) have counterargued that this has historically been more a result of technological restraints than of principled choice. The social dimensions of art is a topic of active interest, which recently inspired a dialogue between philosophers, neuroscientists, artists, and social scientists (Christensen and Gomila, 2018).

Paralleling this conceptual reorientation, certain recent developments in neuroscience have motivated and informed a more nuanced and comprehensive picture of the artistic brain. In particular, the advent of the social brain connectome—a “wiring diagram” of the neural connections that regulate social cognition—has transformed our view of human social behavior as a neurobiological phenomenon (Alcalá-López et al., 2017), demonstrating that distributed and interlocking neural networks support and integrate the diverse processes that together mediate our interactions with other people and their artifacts. This recent paradigm shift in social neuroscience promises to have an equally transformative impact on neuroaesthetics, and our view of art as an construct of the human social brain. There are three main reasons for this. Firstly, demonstrating shared neural circuitry engaged by both art perception and social interaction may illuminate the neural mechanisms that are common to both (Van Leeuwen, 2020). Secondly, establishing the social brain connectome lays the ground for experiments that can assess and visualize the engagement of the social brain by art empirically. Finally, by considering the social context integral to complex behavioral constructs, the emerging paradigm might align neuroaesthetics conceptually with prevailing cultural issues surrounding the nature, value and purpose of art that that have occupied practicing artists over the past century.

Our aims in this review are firstly, to examine the evidence that art engages the social brain; secondly, to show how this neural architecture might operate in viewing artworks; and finally, to work out some neuroscientific and clinical implications raised by our formulation. We start from the hypothesis (widely endorsed by artists themselves) that art is in the first place a social construct, which cannot be divorced from its perceptual and aesthetic qualities: it is always produced and validated within a societal context based on shared cultural values and can only be fully understood as a social object. We further hypothesize that components of the social brain connectome support the analysis, appreciation and behavioral response to artworks. Neuroscience cannot answer what is or what is not (good) art. It can, however, attempt to illuminate how art and creativity relate to other complex human behaviors, and identify factors that tend to promote particular aesthetic valuations. We present a *prima facie* case for visual art as a social brain phenomenon, drawing pre-eminently on evidence derived from the social brain connectome to propose an “artistic brain connectome.” We argue that neuroaesthetics should engage with this evidence, suggest practical and clinical applications of the artistic brain connectome with particular reference to aging and dementia, and outline a roadmap for further experimental work.

## Art in the Social Brain

### The Social Brain Connectome

The social brain connectome or “Social Brain Atlas” was created by Alcalá-López et al. (2017) from the largest coordinate-based quantitative meta-analysis on social cognition to date, comprising 26 meta-analyses encompassing results from 3,972 separate functional magnetic resonance imaging (fMRI) and positron emission tomography (PET) studies, based on data from 22,712 neurologically healthy adults. Before we will describe how we contextualized art in the social brain, we will elaborate briefly on how the social brain connectome was constructed, as this has bearing on the conceptualizations and terminology used when describing the Social Brain Atlas in the following sections.

The meta-analysis on social cognition neuroimaging studies that was performed by Alcalá-López et al. (2017) assessed both task-dependent and task-free (resting-state) studies. The authors defined social cognition as “the processing of information on human individuals, opposed to the aspects of the physical world.” All published meta-analytic review papers related to any type of social-affective cognition were eligible for inclusion. The PubMed data base was searched for quantitative meta-analyses on fMRI and PET studies, using combinations of the following search terms: “social,” “affective,” “emotional,” “face,” “judgment,” “action observation,” “imitation,” “mirror neuron,” “empathy,” “theory of mind,” “perspective taking,” “fMRI,” and “PET.” Further studies were identified through review articles and reference tracing from the retrieved papers. Inclusion criteria for eligible studies were as follows: (1) full brain coverage, (2) absence of pharmacological manipulations, and (3) absence of brain lesions or known mental disorders. Additionally, meta-analytic studies were only considered if they reported (4) convergence locations of whole-brain group analyses as coordinates according to the standard reference space Talairach/Tournoux or MNI (Montreal Neurological Institute). Excluded were experiments assessing neural effects in *a priori* defined regions of interest. Thirty six consensus social brain areas were identified.

For each social brain area that arose from the meta-analysis, Alcalá-López et al. (2017) generated a functional profile, by means of both forward and reverse inference. In forward inference, neural network dynamics are predicted based on theories about the nature of psychological processes, whereas in reverse inference, likely psychological functions are predicted from observed brain area activity during a neuroimaging experiment. For this study, we have only drawn on the functional profiles in the social brain derived at by reverse inference by Alcalá-López et al. (2017), as this covered the broadest range of mental operations and behaviors. Functional annotations in the social brain connectome were assigned by Alcalá-López et al. (2017), using an automated, largescale, multi-dimensional generative framework similar to that described by Yarkoni et al. (2011). The implication of each brain region in particular psychological functions was quantified in the reverse inference analysis, as the probability of a cognitive function term occurring in an article given the documentation of activation in the relevant brain region [P(Term|Activation)]. The taxonomy of the functional neuroanatomical profiles was based on categories of the Behavioral Domain (mental operations) and Paradigm Class (experimental tasks and stimuli), derived from the BrainMap taxonomy (BrainMap, 2003).

### Mapping Art Processing Onto the Social Brain Connectome

#### Derivation of the “Artistic Brain Connectome”

Here we applied qualitative reverse inference to the social brain connectome, in order to map candidate cognitive processes engaged by visual art and visuospatial creativity onto the functional neuroanatomical profiles that are most likely to underpin them. We broadened the definition of “social cognition” offered by Alcalá-López et al. (2017), from “the processing of information on human individuals, opposed to the aspects of the physical world” to “the processing of information that is necessarily grounded in the social context a subject operates in, regardless of whether the information is generated internally or externally.” The taxonomy we used to describe experimental tasks and mental operations related to art and visuospatial creativity is based on the descriptions that were used in the reviewed papers. The PubMed database was searched using combinations of the term “visual art” with “brain”; “neuroaesthetics”; “colors”; “visuospatial creativity”; “fMRI”; “PET” and “MEG”. Since our aim was to provide a proof of principle, we focused mostly on seminal neuroimaging studies on visual art and visuospatial creativity that have been published in the field of neuroaesthetics. In our literature review we included studies that involved evaluative neuroaesthetic tasks (e.g., making perceptual judgments about artworks) and/or contemplative tasks (e.g., reflecting on the personal meaning of artworks). While the mode of engagement with art is a fundamental consideration in neuroaesthetic study design, and while these modes are separable and likely to be mediated by partly separable neural mechanisms, they are not mutually exclusive. We emphasize that both modes of viewing are often engaged when we encounter artworks in the world at large—and further, their component cognitive processes are supported by closely interacting neural networks. We excluded neuroimaging studies that treated visual art simply as a special category of experimental stimuli, whose visual properties were manipulated to make inferences about their perceptual, emotional or cognitive effects.

Table 1 summarizes key brain areas derived from the social brain connectome in the Social Brain Atlas, alongside the results of our analysis proposing art processing functions principally mediated by each of these areas. Together, these constitute the “artistic brain connectome.” The different subparts of the Table (Table 1(A)… etc.) focus on each of the large-scale functional networks that comprise the social brain connectome, and our interpretation of these networks according to their putative roles within the artistic brain connectome.

**TABLE 1:** Summary of social and proposed art processing functions mediated by the social brain connectome.

(A) Level 1: Perceptual analysis of art and expressions of creativity		

Social brain area	Social brain atlas functional profiles	Art processing functions

Level 1	Lower sensory network	Perception network
pSTS_R	2(Cognition, Memory Working, Language Semantics and Speech); 1(Attention, Emotion, Music Comprehension/Production, Word Generation (Covert), Reward)	Evaluation of expressiveness of portraits
pSTS_L	3(Memory Working); 2(Language Semantics); 1[Emotion, Language Speech, Music Comprehension/Production, Passive Listening, Reward, Word Generation (Covert)]	
FG_R	2(Emotion); 1(Action Execution, Audition, Cognition, Language Semantics, Memory Explicit, Memory Working, Reward, Vision Shape, Visuospatial Attention)	(Naturally colored) Objects and faces representations in pictures
FG_L	2(Language Semantics, Vision Shape); 1(Attention, Audition, Cognition, Emotion, Face Monitoring, Finger Tapping, Language Speech, Memory Explicit and Working, Passive Listening and Viewing, Reward)	(Naturally colored) Objects and faces representations in pictures
MTV5_R	2(Emotion, Language Semantics); 1(Face Monitoring, Language Speech, Memory Explicit and Working, Music Comprehension/Production, Reward, Social Cognition, Vision Motion)	Implied motion perception in pictures
MTV5_L	2(Language Semantics and Speech, Reward); 1(Action Execution, Attention, Cognition, Emotion, Emotion Induction, Memory Explicit and Working, Passive Listening, Vision Shape)	Implied motion perception in pictures

**(B) Level 2: Animating dynamics of art and creativity**		

**Social brain area**	**Social brain atlas functional profiles**	**Art processing functions**

**Level 2**	**Limbic network**	**Animation network**

vmPFC	4(Emotion); 2(Memory Working); 1(Action Execution, Attention, Cognition, Imagined Objects/Scenes, Language Semantics, Passive Listening, Reasoning, Social Cognition)	(Naturally colored) Objects representations in pictures, personal reward value of art
rACC	3(Emotion); 2(Memory Working, Reward); 1(Cognition, Face Monitoring, Fear, Language Semantics and Speech, Memory Explicit, Reasoning)	
NAC_R	4(Emotion, Memory Working); 1(Language Semantics)	
NAC_L	4(Emotion, Memory Working); 1(Cognition, Emotion Induction, Language Syntax, Reward)	
AM_R	4(Emotion); 2(Language Speech, Reward); 1(Action Execution, Audition, Cognition, Face Monitoring, Finger Tapping, Memory Working)	Emotion induction by art, face representations in pictures
AM_L	3(Memory Working); 1(Action Execution, Cued Explicit Recognition, Emotion, Face Monitoring, Finger Tapping, Language Semantics and Speech, Memory Explicit)	Emotion induction by art, face representations in pictures
HC_R	3(Memory Working); 2[Cognition, Emotion, Language Semantics, Word Generation (Overt)]; 1(Face Monitoring, Imagined Objects/Scenes, Mental Rotation)	Naturally colored objects and landscape representations in pictures, visuospatial creative production
HC_L	3(Emotion); 2(Face Monitoring, Language Semantics, Memory Working, Reward); 1(Cognition, Finger Tapping, Language Speech, Word Generation (Overt)	Naturally colored objects in pictures, personal resonance with art, visuospatial creative production

**(C) Level 3: Interactive significance of art and creativity**		

**Social brain area**	**Social brain atlas functional profiles**	**Art processing functions**

**Level 3**	**Intermediate network**	**Interaction network**

IFG_R	2(Emotion); 1(Attention, Audition, Cognition, Face Monitoring, Language Semantics and Speech, Reward)	Visuospatial creative ability; increased connectivity with default mode network
IFG_L	3(Emotion); 2(Language Semantics, Memory Explicit); 1(Attention, Audition, Face Monitoring, Finger Tapping, Language Speech, Memory Working, Passive Listening, Vision Shape)	Verbal creative ability; increased connectivity with default mode network
aMCC	2(Cognition, Emotion, Reward); 1(Anxiety, Attention, Language Semantics and Speech, Memory Working)	Viewing pictures with unusually colored objects, critical evaluation of aesthetic experience and creative thoughts
AI_R	3(Action Execution); 2(Attention, Memory Working, Reward); 1(Audition; Cognition, Emotion, Face Monitoring, Finger Tapping, Memory Explicit, Passive Listening)	Evaluation of Artwork Brightness and Aesthetic Value
AI_L	2(Cognition, Language Semantics); 1(Action Execution and Inhibition, Audition, Emotion, Emotion Induction, Finger Tapping, Reasoning, Reward)	Evaluation of artwork brightness and aesthetic value, dynamic divergent/convergent creative thoughts switching
SMA_R	2(Emotion); 1(Action Execution, Attention, Cognition, Face Monitoring, Memory Explicit and Working, Passive Listening, Reward)	Evaluation of artwork brightness and aesthetic value
SMA_L	2(Action Execution, Memory Working); 1(Attention, Audition, Cognition, Emotion, Face Monitoring, Finger Tapping, Language Semantics, Memory Explicit, Passive Listening, Reward, Vision Shape)	Evaluation of artwork brightness and aesthetic value
pSTS_R	2(Cognition, Memory Working, Language Semantics and Speech); 1(Attention, Emotion, Music Comprehension/Production, Word Generation (Covert), Reward)	Evaluation of expressiveness of portraits
pSTS_L	3(Memory Working); 2(Language Semantics); 1[Emotion, Language Speech, Music Comprehension/Production, Passive Listening, Reward, Word Generation (Covert)]	
SMG_R	2(Emotion, Language Semantics, Memory Working); 1(Action Execution, Action Imagination, Attention, Emotion Induction, Finger Tapping, Language Speech, Passive Viewing, Pitch Discrimination, Vision Shape, Word Generation (Covert)	
SMG_L	2(Audition, Language Semantics); 1(Action Inhibition, Emotion, Memory Explicit and Working, Reward, Vision)	Thought generation for novel object uses

**(D) Level 4: Symbolic and personal meaning of art and creativity**		

**Social brain area**	**Social brain atlas functional profiles**	**Art processing functions**

**Level 4**	**High associative network (corresponds with default mode network)**	**Construction network**

mFP	3(Emotion); 2(Happiness); 1[Face Monitoring, Fear, Language Semantics, Memory Working, Music Comprehension/Production, Sadness, Social Cognition, Word Generation (Covert)]	Authenticity evaluation of art
dmPFC	3(Emotion); 2(Mental Rotation, Memory Working, Reward); 1(Action Execution, Cognition, Fear, Language Orthography, Music Comprehension/Production, Sadness, Social Cognition)	Personal resonance with art, creative thought generation, creative production
TP_R	3(Reward); 2(Action Execution, Cognition, Emotion, Happiness, Reasoning, Social Cognition); 1(Attention, Audition, Emotion Induction, Face Monitoring, Language, Memory Working, Music Comprehension/Production, Vision Motion)	Conforming art evaluation and creative production to social norms and values
TP_L	3(Emotion); 2(Audition, Cognition, Reward); 1(Action Execution and Observation, Attention, Happiness, Language Semantics, Mental Rotation, Music Comprehension/Production, Reasoning)	Conforming art evaluation and creative production to social norms and values
MTG_R	3(Memory Working); 2[Emotion, Language Semantics, Music Comprehension/Production, Word Generation (Overt)]; 1[Action Inhibition, Cognition, Happiness, Language, Language Speech, Vision, Visuospatial Attention, Rewards, Social Cognition, Word Generation (Covert)]	Creative thought generation, creative production
MTG_L	2(Emotion, Language Semantics); 1(Action Execution, Cognition, Language Orthography and Speech, Mental Rotation, Memory Working, Music Comprehension/Production, Social Cognition, Vision Shape)	Creative thought generation, creative production
pMCC	3(Emotion, Memory Working); 1(Action Execution, Action Inhibition, Disgust, Fear, Language Semantics, Visuospatial Attention)	
TPJ_R	2(Emotion); 1(Action Execution and Observation, Cognition, Language Semantics, Memory Working, Passive Listening, Reward, Social Cognition, Space)	Figurative representations in paintings
TPJ_L	2(Emotion, Cognition, Language Semantics, Memory Working, Reward); 1(Language Speech, Memory Explicit, Music Comprehension/Production, Vision Motion, Visual Object Identification)	
PCC	3(Memory Working); 2(Emotion, Cognition, Face Monitoring, Reward); 1(Language Semantics and Speech, Music Comprehension/Production)	Personal and symbolic meaning of art, creative thought generation, creative production
Prec	2(Emotion, Memory Working, Reward); 1(Action Execution, Attention, Cognition, Language Semantics and Speech, Music Comprehension/Production, Sadness, Social Cognition, Space)	Visuospatial qualities of visual artworks, creative thought generation, creative production

*Terms for psychological processes in column 2 are derived from the Behavioral Domains of BrainMap. Participation of particular brain regions in different psychological processes has been quantified using reversed inference (Alcalá-López et al., 2017), based on the following likelihood ratios: 1() = 4–9.9; 2() = 10–19.9; 3() = 20–29.9; 4() = 30–38. BrainMap uses a structured standardized coding scheme to describe published human neuroimaging experimental results, with the goal to facilitate the development of software and tools to share neuroimaging results and enable meta-analysis of studies of human brain function and structure in healthy and diseased subjects. This might explain why the functional profiles of the Social Brain Atlas appear closely aligned with the syntax of programming languages and can at times strike as agrammatical English or at odds with common terminology used by clinicians to describe cognitive functions. Art processing functions have been attributed qualitatively. Social brain connectome abbreviations in alphabetical order: AI, Anterior insula; AM, Amygdala; aMCC, Anterior mid-cingulate cortex; Cereb Cerebellum; dmPFC, Dorsomedial prefrontal cortex; FG, Fusiform gyrus; HC, Hippocampus; IFG, Inferior frontal gyrus; mFP, Medial frontal pole; MTG, Middle temporal gyrus; MT/V5, Middle temporal V5 area; NAC, Nucleus accumbens; PCC, Posterior Cingulate Cortex; pMCC, Posterior mid-cingulate cortex; Prec, Precuneus; pSTS, Posterior superior temporal sulcus; rACC, Rostral anterior cingulate cortex; SMA, Supplementary motor area; SMG, Supramarginal gyrus; TP, Temporal pole; TPJ, Temporo-parietal junction; vmPFC, Ventromedial prefrontal cortex. An extension with _L indicates “Left,” while an extension with _R indicates “Right.”*

#### Perceptual Analysis of Art

**FIGURE 1:**
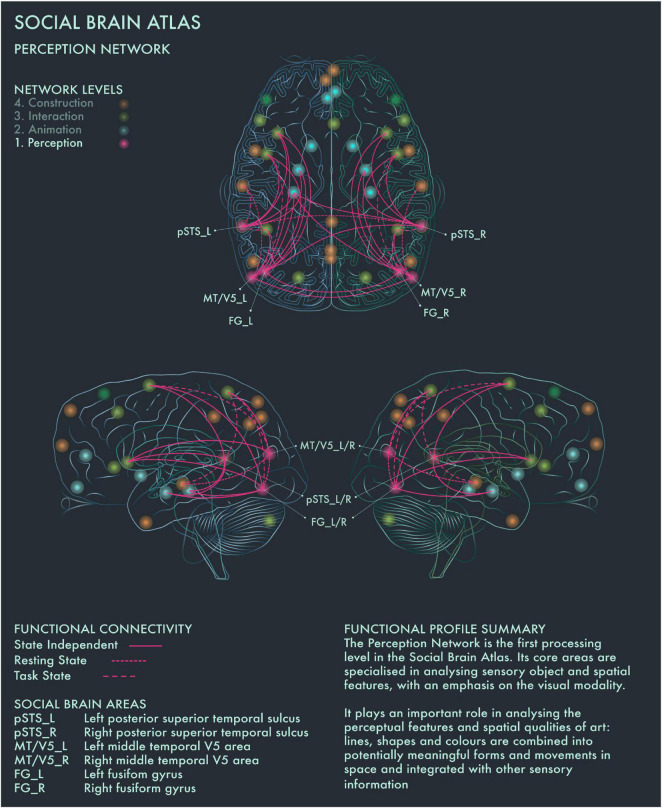
Social Brain Atlas Level 1: Perception Network. This figure shows the functional connectivity patterns of the Perception Network (PN), based on data derived from Alcalá-López et al. (2017) (see also Table 1(A)). The core nodes of the PN have been labeled as follows: Bilateral posterior superior temporal sulcus (pSTS_L/R); Bilateral middle temporal V5 area (MT/V5_L/R); Bilateral fusiform gyrus (FG_L/R). Areas beyond the PN to which these core nodes project are not labeled. Functional connections have been drawn as lines; a solid line indicates a functional connection independent of the brain state, a wide dotted line indicates a task-dependent functional connection and a narrow-dotted line indicates a functional connection during a brain state with no output task (the resting state). The PN is the first processing level in the social brain connectome Its core areas are specialized in analyzing sensory object and spatial features, with an emphasis on the visual modality. It plays an important role in analyzing the perceptual features and spatial qualities of art: lines, shapes and colors are combined into potentially meaningful forms and movements in space and integrated with other sensory information.

The first processing level of the social brain connectome, designated the “Lower Sensory Network” by Alcalá-López et al. (2017), is likely to play an important role in the perceptual analysis of object features (colors and forms) and spatial relationships in visual art as well as social scenes more generally. With respect to art processing, we designate it the Perception Network (Figure 1).

Cela-Conde et al. (2013) found that within 750 ms after the presentation of a visual stimulus, the brain makes an assessment whether it is beautiful or not. The authors proposed to call this phase the “Initial Aesthetic Network,” which covers occipital, temporal and parietal areas. The connectivity patterns of this network correspond with both the ventral (lower) and dorsal (upper) streams of visual processing. The ventral stream is concerned with the “What?” of visual information to guide the meaning making process, whereas the dorsal stream is focused on the “Where?” to guide action (Ungerleider and Mishkin, 1982; Goodale and Milner, 1992; Ungerleider and Haxby, 1994). Recruitment of bilateral fusiform gyrus (FG) has been associated with object representation (in particular, faces) when viewing pictorial representations (Kawabata and Zeki, 2004; Vartanian and Skov, 2014). Both the left and right FG are associated with shape vision and the right FG is likely to be activated during visuospatial attention (Alcalá-López et al., 2017). Photographic images of naturally and unnaturally colored objects have been found to activate areas V1–V4 of the visual cortex in equal manner. However, images of naturally colored objects have been reported to engage more with the ventral stream, including the anterior regions of the fusiform gyrus (Zeki and Marini, 1998). The perception of implied motion in paintings has been associated with increased activity of canonical visual motion processing areas in bilateral middle temporal V5 (MT/V5) cortices. Cattaneo et al. (2017) found that area V5 repression using transcranial magnetic stimulation reduced motion perception in both figurative and abstract paintings in art-naïve viewers. Conversely, activation of MT/V5 areas is associated with perception of motion in abstract art, but only in viewers who had previous experience detecting motion in abstract paintings (Kim and Blake, 2007). Bilateral MT/V5 areas are further implicated in “working memory” and “explicit memory” tasks (Alcalá-López et al., 2017). Posterior superior temporal sulcus (pSTS) is part of both the Perception and the higher-order Interaction Network and is the only area in the social brain connectome that is clustered within two different network profiles: this may reflect its role in linking bottom-up sensory information with top-down interpretative processes during evaluations of the expressiveness of portraits (Ferrari et al., 2018).

#### Animating Dynamics of Art

**FIGURE 2:**
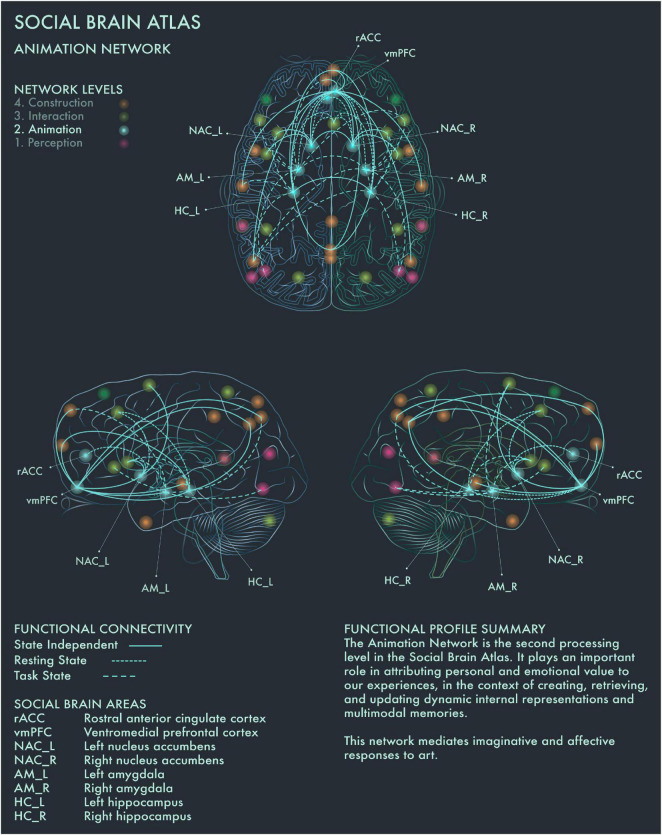
Social Brain Atlas Level 2: Animation Network. This figure shows the functional connectivity patterns of the Animation Network (AN), based on data derived from Alcalá-López et al. (2017) (see also Table 1(B)). Graphical conventions are the same as Figure 1. The core nodes of the AN have been labeled as follows: Rostral anterior cingulate cortex (rACC); Ventromedial prefrontal cortex (vmPFC); Bilateral nucleus accumbens (NAC_L/R); Bilateral amygdala (AM_L/R); Bilateral hippocampus (HC_L/R). The AN is the second processing level in the social brain connectome. It plays an important role in attributing personal and emotional value to our experiences, in the context of creating, retrieving, and updating dynamic internal representations and multimodal memories. This network mediates imaginative and affective responses to art.

The second processing level of the social brain connectome, designated the Limbic Network by Alcalá-López et al. (2017), mediates emotional responses, reward, learning and dynamic spatial representations. In the context of art processing and creativity, we have designated this network the “Animation Network” (Figure 2). Animation is derived from the Latin *animare*, meaning “to endow with a particular spirit, to enliven.” We believe that “animation” aptly captures both the important role this network plays in the affective components of art perception and creation, as well as its role in dynamic object and scene constructions during visuospatial creative thought processes.

Amygdala (AM) plays a crucial role in emotion coding during reward attribution, including aesthetic evaluations (Cela-Conde et al., 2013). Bilateral AM has been found to be specifically engaged by viewing portrait paintings, as well as faces more generally (Kawabata and Zeki, 2004). Left hippocampus (HC) has been found to be engaged when viewing highly moving artworks (Vessel et al., 2012), while selective activation of the right HC has been reported when viewing landscape paintings (Kawabata and Zeki, 2004). Bilateral HC activation occurs during visuospatial creative production (Kowatari et al., 2009; Ellamil et al., 2012), while bilateral HC damage associated with anterograde amnesia leads to difficulty imagining scenes and objects (Hassabis et al., 2007; Mullally et al., 2012). Bilateral HC has also been found to be activated when viewing pictures of naturally, but not unnaturally, colored objects (Zeki and Marini, 1998). Within the social brain connectome, HC is functionally connected during resting state with anterior mid-cingulate cortex (aMCC) in the intermediate network, allowing for integration of affective and semantic evaluation of aesthetic experience in art. Ventromedial prefrontal cortex (vmPFC) is the end-point of the ventral visual pathway (Goodale and Milner, 1992; Ungerleider and Haxby, 1994) and is involved when viewing images of naturally colored images as well as in evaluating the potential reward value of a visual experience (Elliott et al., 2000). It has been implicated more particularly in the aesthetic appraisal of artworks (Cela-Conde et al., 2004, 2013; Kawabata and Zeki, 2004; Ishizu and Zeki, 2011, 2013; Lacey et al., 2011). This is congruent with the functional profile of this region in the Social Brain Atlas, which implicates it in emotion, attention, reward processing, social decision making and imagined objects/scenes and reasoning (Alcalá-López et al., 2017). In summary, the Animation Network is likely to be integral to the (aesthetic) evaluative stance toward artworks, although conflicting findings have been reported regarding whether this is a spontaneous or intentional process (Höfel and Jacobsen, 2007; Kreplin and Fairclough, 2013).

#### Interactive Significance of Art

The third processing level of the social brain connectome, designated the Intermediate Network by Alcalá-López et al. (2017), plays an important role in mediating between incoming, potentially significant sensory information and internal states and goals. This network contains core areas [in particular, anterior insula (AI) and anterior mid-cingulate cortex (aMCC)] of the Salience Network, which weighs the significance and relevance of incoming sensory information against current homeostatic priorities in regulating social behavior (Seeley et al., 2007).

**FIGURE 3:**
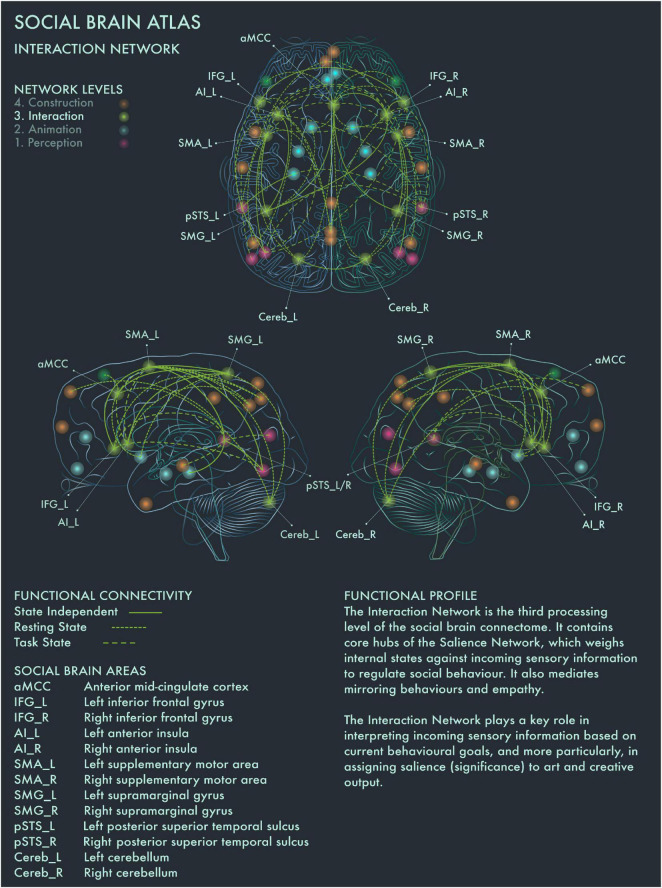
Social Brain Atlas Level 3: Interaction Network. This figure shows the functional connectivity patterns of the Interaction Network (IN), based on data derived from Alcalá-López et al. (2017) (see also Table 1(C)). Graphical conventions are the same as Figure 1. The core nodes of the IN have been labeled as follows: Anterior mid-cingulate cortex (aMCC); Bilateral inferior frontal gyrus (IFG_L/R); Bilateral interior insula (AI_L/R); Bilateral supplementary motor area (SMA_L/R); Bilateral supramarginal gyrus (SMG_L/R; Bilateral posterior superior temporal sulcus (pSTS_L/R); Bilateral cerebellum (Cereb_L/R). The IN is the third processing level of the social brain connectome. It contains core hubs of the Salience Network, which weighs internal states against incoming sensory information to regulate social behavior. It also mediates mirroring behaviors and empathy. The IN plays a key role in interpreting incoming sensory information based on current behavioral goals, and more particularly, in assigning salience (significance) to art and creative output.

This network is therefore likely to be integral to the construction of significance in art, based on the perceived salience of artworks. Artworks tend to be highly valued in themselves, invested with emotional and cultural associations and often encountered under conditions of “ceremony” and heightened expectation; accordingly, they tend to be salient stimuli for many viewers. However, the salience of a particular artwork is heavily modulated by prior personal familiarity (with the artwork in question and its genre more widely), the socio-emotional context in which we view it and our behavioral stance toward it. Salience Network regions have functional connections to vmPFC and other core nodes from the Limbic (Animation) Network, enabling affective modulation of salience coding. Taken together, with respect to art processing and visuospatial creativity, this network appears to play a key role in deciding on whether to engage deeper or whether to disengage from an artwork, and we therefore designate this the Interaction Network (Figure 3).

Bilateral AI and supplementary motor area (SMA) are engaged when making judgments about the brightness and aesthetic value of paintings (Ishizu and Zeki, 2013). Cela-Conde et al. (2013) reported that left AI mediates between the preliminary appraisal of artworks and deeper processing (reflected by engagement of the Default Mode Network) if the artwork has been judged as beautiful. aMCC is activated by pictures of unnaturally colored objects (Zeki and Marini, 1998) and also during critical evaluation of aesthetic experience (Kawabata and Zeki, 2004; Boccia et al., 2016) and creative production (Ellamil et al., 2012; De Pisapia et al., 2016; Beaty et al., 2018). Left supramarginal gyrus (SMA) has been associated with the generation of ideas for novel object uses (Benedek et al., 2018). Left AI has been implicated more specifically in dynamic switching between divergent and convergent thought processes, a key neural mechanism underlying creativity (Ellamil et al., 2012; De Pisapia et al., 2016; Beaty et al., 2018), while creative ability has been linked to increased functional connectivity between bilateral IFG and DMN during creative thought generation (Kowatari et al., 2009; Ellamil et al., 2012; Beaty et al., 2014, 2018; De Pisapia et al., 2016). These findings are congruent with the functional profiles of AI, aMCC, SMA and IFG in the social brain connectome, which implicate these regions in a range of cognitive functions related to action execution and inhibition (Alcalá-López et al., 2017). Recruitment of left IFG was reported for “vision shape” tasks by Alcalá-López et al. (2017) and may indicate its engagement in visuospatial behavior is modulated by specific stimulus characteristics or task demands. The emerging picture suggests that the Interaction Network plays an important role in mediating between evaluative and contemplative responses to art.

#### Symbolic and Personal Meaning of Art

The fourth processing level of the social brain connectome, designated the High Association Network by Alcalá-López et al. (2017), is engaged in creating symbolic models of the outside world and interpreting and responding to mental states of self and others. This network encompasses core components of the Default Mode Network (DMN) and semantic appraisal networks. DMN hosts a dynamic interface between the contents of the self-schema (homeostatic signals, mental states and memories) and the world at large, including in particular how oneself relates to other people (Buckner et al., 2008).

**FIGURE 4:**
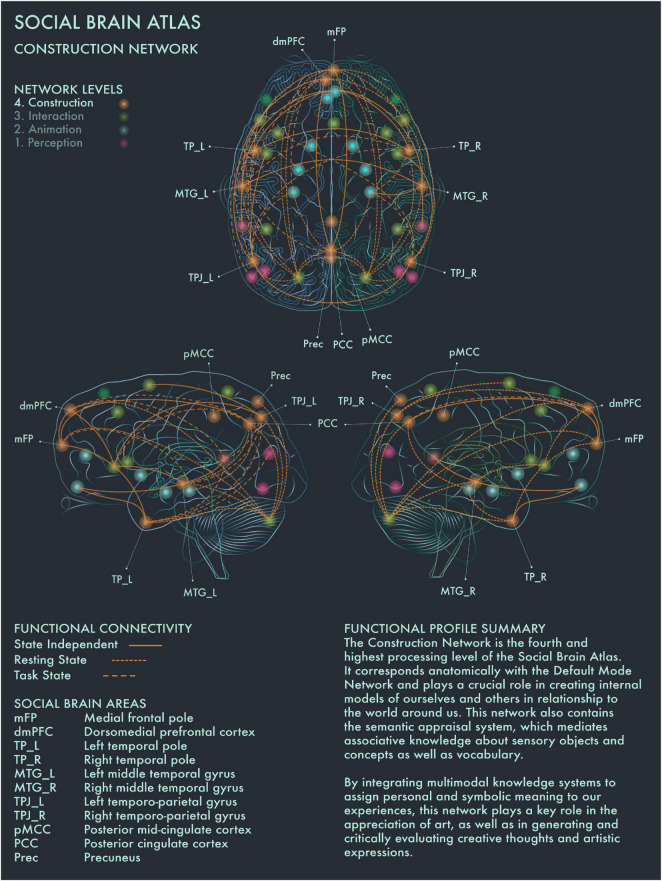
Social Brain Atlas Level 4: Construction Network. This figure shows the functional connectivity patterns of the Construction Network (CN), based on data derived from Alcalá-López et al. (2017) (see also Table 1(D)). Graphical conventions are the same as Figure 1. The core nodes of the CN have been labeled as follows: Medial frontal pole (mFP); Dorsomedial prefrontal cortex (dmPFC); Bilateral temporal pole (TP_L/R); Bilateral middle temporal gyrus (MTG_L/R); Bilateral temporo-parietal junction (TPJ_L/R); Posterior mid-cingulate cortex (pMCC); Posterior Cingulate Cortex (PCC); Precuneus (Prec). The CN is the fourth and highest processing level of the social brain connectome. It corresponds anatomically with the Default Mode Network and plays a crucial role in creating internal models of ourselves and others in relationship to the world around us. This network also contains the semantic appraisal system, which mediates associative knowledge about sensory objects and concepts as well as vocabulary. By integrating multimodal knowledge systems to assign personal and symbolic meaning to our experiences, the CN has a key role in the appreciation of art, as well as in generating and critically evaluating creative thoughts and artistic expressions.

This network is therefore well placed to code and to verbalize the symbolic meaning of and personal resonance with art: how one’s own mental and homeostatic states are being impacted by the mental states (as expressed in artworks) of other people. Such processing might be particularly to the fore during “contemplative” engagement with artworks. However, this is likely to be a highly active process: unlike basic emotions, deciphering the personal and symbolic meaning of an artwork is ambiguous and full of uncertainty, requiring an imaginative entry into (or “theory of”) the mind of the artist as well as an open-minded exploration of the symbolism communicated by the artwork (Nadal and Chatterjee, 2019). Also integral to interpreting the actions, artifacts, and symbols generated by other people, is stored knowledge (verbal and non-verbal “lexicons”) about the world, mediated by the semantic appraisal network anchored in bilateral temporal pole (TP) (Mummery et al., 2000). TP atrophy in neurodegenerative disease has been associated with the development of obsessive interest in music (musicophilia), bright colors, and highly idiosyncratic visuospatial creative production (Fletcher et al., 2013; Erkkinen et al., 2018). This suggests TP has a modulating role in art evaluation and creative production, which holds personal desires and expressive visions in check to conform to social norms and values (Erkkinen et al., 2018). With respect to art processing and visuospatial creativity, we designate this the Construction Network, to emphasize its role in constructing the (inter-) personal and symbolic meaning of art and creative processes (Figure 4).

DMN has been reported to be engaged when people are strongly moved by an artwork, and also when people perceive an artwork as beautiful (Vessel et al., 2012, 2013; Cela-Conde et al., 2013). Cela-Conde et al. (2013) proposed that this recruitment of the DMN during art viewing represents the “Delayed Aesthetic Network,” during which the experience of beauty becomes conscious. When an artwork is perceived as “ugly,” internal attention is redirected instead (Cela-Conde et al., 2013). It has been suggested that DMN dynamics track people’s internal state during aesthetic experiences and that engagement of DMN components regulates aesthetic orientation across the visual domain (Chatterjee and Vartanian, 2014; Belfi et al., 2019; Vessel et al., 2019). Posterior cingulate cortex (PCC) has been found to be engaged when people imbue an artwork with personal and symbolic meaning, regardless of its artistic category or style (Boccia et al., 2016). PCC is highly co-activated with dorsomedial prefrontal cortex (dmPFC), bilateral middle temporal gyrus (MTG) and other social brain networks in divergent and creative thought and production (Ellamil et al., 2012; Gonen-Yaacovi et al., 2013; Beaty et al., 2014, 2018; De Pisapia et al., 2016). Right TPJ showed greater activation when viewing representational than intermediate or abstract paintings (Fairhall and Ishai, 2008). Precuneus (Prec) has been implicated in exploring the visuospatial qualities of visual artworks (Kawabata and Zeki, 2004; Fairhall and Ishai, 2008; Cupchik et al., 2009; Vartanian and Skov, 2014; De Pisapia et al., 2016). Activation of frontal pole (FP) is engaged during evaluation of the authenticity of paintings, especially when the artwork is considered potentially inauthentic (Huang et al., 2011). The distributed functional connections of medial FP to multiple other areas in the social brain connectome with strong links to emotion, working memory and reward processing would support a critical role for this region in social and cultural value assessments.

#### Putting the Artistic Brain Connectome Together

Empirical support for extensive cross-talk between these different processing levels of the social brain comes from research by Housen (1987, 1999, 2002) in the context of art interactions, which found that people’s perception and appreciation of art is strongly filtered by their internally constructed models of how art relates to the social world. Personal values and internally constructed world models are reflected in the way individuals respond to art. When an individual feels a strong personal connection when engaging with art, the Default Mode Network (DMN) is co-activated dynamically with other large-scale brain networks (Cela-Conde et al., 2004; Fox et al., 2005; Ishizu and Zeki, 2011; Vessel et al., 2012; Cela-Conde et al., 2013; Ishizu and Zeki, 2013; Vessel et al., 2013; Vartanian and Skov, 2014). This coupling is thought to occur when integration of different memory modalities is required for optimal mental functioning, and has been found to play an important role in self-relevant and social, as well as creative thought processes (Amodio and Frith, 2006; Spreng and Grady, 2010; Sestieri et al., 2011; Ellamil et al., 2012; Beaty et al., 2014, 2018; Chatterjee and Vartanian, 2014; Margulies et al., 2016; Alcalá-López et al., 2017).

### The Artistic Brain Connectome in Operation

#### Predicting and Analyzing the Social Brain Dynamics of Artwork Encounters

How is the social brain connectome engaged when we encounter works of art? Based on the picture emerging from the foregoing literature review, we argue that encounters with artworks entail four processing levels, corresponding to the component neural networks of the “artistic brain connectome” as we have outlined them here. We do not wish to imply that neural processing of an artwork proceeds linearly through these four levels; under most circumstances when we view art in everyday life, information will be mutually exchanged among levels (processing networks) and the viewer’s perspective will shift between contemplative and evaluative modes of engagement. As is clear from the evidence reviewed above, the circuit organization of the artistic brain connectome allows for information transfer in parallel and reciprocally as well as hierarchically between levels, influenced by bottom-up and top –down influences that extend across levels.

1. **Perceptual analysis (Perception Network).** This process is generic to a broad range of complex visual phenomena but the coding of featural and spatial relations between constituents of a particular visual artwork and relations between artworks in a physical space have analogies to parsing complex social “scenes”—especially the coding of (potential) sensory ambiguity and incongruity, which are integral both to art and inter-personal interactions.
2. **Animating dynamics (Animation Network).** Artworks, like people, are highly affectively laden sensory objects and demand a “creative” orientation: i.e., the imagining and selection of potential responses, modulated by prior familiarity and potential emotional and reward value. This processing is rapid and intrinsically active (since artworks and persons generally must be processed in a context or “scene” that is assigned by the perceiver based on previously experienced similar spatiotemporal configurations), dynamic (since emotional and hedonic context is frequently in flux) and entails novelty (since our responses to artworks, as to other people, are challenging to predict *a priori*). This processing is also integral to our subjective “aesthetic sense” when engaging with art.
3. **Interactive significance (Interaction Network**). How a viewer engages with an artwork depends fundamentally on stored norms—concepts and “rules” derived implicitly through accumulated past experience of the art of a culture—as well as its perceived beauty and the viewer’s own inner homeostatic state and current behavioral priorities. Analogous operations are engaged when we comprehend and evaluate the behavioral signals of other people. Norm conformation and violation determine the relative “salience” of an artwork viewed among other artworks (or non-artistic objects); and salience coding by the Interaction Network is in turn integral to our affective and symbolic appraisal of artworks as well as artistic creativity.
4. **Symbolic and personal meaning (Construction Network**). Artworks, like people, convey states of mind that must be decoded. In addition to the mental states represented by artworks in themselves, they embody the intent of the artist in creating them. Interpreting these mental states is integral to the appreciation of meaning in any artwork and is likely to engage neural operations that mediate’ theory of mind’, by constructing a mental model or narrative of others’ mental states: a core process of social cognition. Indeed, art could be considered a window on the brain’s internally generated models of feeling states.
5. **Generation of an integrated response (Cross-Network).** An individual’s personal engagement with and appreciation of the “meaning” of an artwork are likely to depend on a complex and dynamic interplay between past experience, expectation and novelty (“surprise”) (see Box 1). These factors will also affect the pleasure the individual takes in the artwork. The individual’s final cognitive and affective response to the artwork will depend on integrated neural activity across the artistic brain connectome.

**Box 1. The artistic brain connectome encounters an artwork.**

Here we consider how the artistic brain connectome might be engaged in the viewer’s response to a particular artwork. We take as our example David Hockney’s iPad drawing No. 2 from “The Arrival of Spring in Woldgate, East Yorkshire,” 2011. We chose this example as a mainstream artwork whose subject (a quotidian landscape scene) is not explicitly “social” or inter-personal, to illustrate the generalizability of the social brain hypothesis of art processing. This work can be viewed on the official David Hockney webpage (Url retrieved on 31-01-2022): https://www.hockney.com/index.php/works/digital/arrival-of-spring-woldgate

Consider the hypothetical case of two friends, A and B, who encounter the drawing for the first time on a visit to the National Gallery in London. A prefers representational to abstract art, and is intimately familiar with Hockney’s art; B on the other hand, does not know who Hockney is, and prefers traditional to contemporary art media. When A and B view the drawing, its visual properties are processed by the Perception Network in both brains; A and B may view the different parts of the image in different order and for varying durations but both will probably conclude that the drawing depicts a country lane in a palette dominated by yellows, pinks and greens mounted on an 11 inch digital tablet, and that the color scheme and the medium are unconventional, relative to the more traditional oil paintings that flank it. However, rapid engagement of the Animation Network generates an initial signal of familiarity and pleasure in A (based on her previous exposure to the artist) but a signal of novelty and potential norm violation in B (who has not encountered the artist previously).

The confirmation or violation of artistic expectation or norms signaled by the initial perceptual analysis is processed in the Interaction Network in each viewer’s brain: this network codes the artwork as a salient emotional sensory object. In the case of A, this may promote “approach” and engagement of the Construction Network, which leads A to form a “theory of mind” about the feeling state embodied in the artwork and the artist’s intent in creating it. Drawing on A’s knowledge of Hockney’s artistic oeuvre, she looks for cues in the drawing that might signify what the artist was attempting to express and the feeling states he has accessed in creating it, while at the same time registering her own past personal experience of the artist’s works. She may recognize the unnatural colors of the landscape as a signature artistic tool used by Hockney to bring out emotional dimensions of the environment, and make people see familiar scenes with different eyes. At the same time, this foregrounding of the artwork’s emotional resonance heightens her awareness of her own emotional responses to the drawing. Although such theory of mind processing is driven by the Construction Network, it is likely to involve a close interaction among all four hierarchical social brain networks. In B, on the other hand, salient norm violation may promote “avoidance,” in which case the Construction Network is not deeply engaged and instead of examining closer what the drawing communicates to her, she moves on to the next artwork.

Note that in our illustrative example we have deliberately and grossly over-simplified the processing dynamics that are likely to occur in each viewer’s artistic brain connectome when viewing an artwork such as this Hockney drawing. In particular, information transfer between the component neural networks is likely to be reciprocal and iterative. However, this example hints at the richness and diversity of our individual encounters with artworks in the world at large. We argue that this complexity and its component processes have formal analogs to our encounters with other people and depend on similar neural mechanisms. Studying how people engage with art, in real-world and laboratory settings might therefore further illuminate the neural dynamics of social behavior.

To the extent that an artwork presents the brain with a “puzzle,” then solving the puzzle may be a source of pleasure—but both willingness to pursue the “solution” and the hedonic charge associated with finding it will be influenced by the viewing context and prior experience and knowledge. People with little experience of viewing art will enter an artwork purely from their own frame of reference of the world as they know it, judging the value of an artwork based on their immediate emotional responses and understanding of what art “should” look like. If a work clashes with the viewer’s expectations, if they cannot detect skill, utility, or purpose, or if they find the subject controversial, then such viewers may devalue the work or be alienated by it. In contrast, experienced art viewers tend to combine a personal with a more universal perspective—when evaluating an artwork, such viewers combine critical skills with their feelings and intuitions to let symbolic meanings emerge. These viewers are in turn likely to be more open to re-evaluate their initial impressions and approach each encounter with an artwork as an opportunity to reflect and gain new insights (Housen, 1987, 1999, 2002).

The viewer’s social environment will importantly shape viewing context, the acquisition of knowledge about art and the “puzzle solving” strategies they bring to the viewing process. The “social constructivist” perspective of Vygotsky may be relevant here. According to this perspective (Vygotsky, 1978), the guidance and collaboration of more capable peers or mentors are instrumental both in the individual’s acquisition of problem-solving competence and in priming their inherent receptivity to a wider range of challenging, unconventional or “problematic” experiences (such as artworks). This constructivist perspective is in line with the “stopping for knowledge hypothesis” proposed by Sarasso et al. (2020), which postulates that aesthetic experiences guide perceptual learning and increase tolerance of uncertainty. However, for any viewer, the degree to which they are willing and able to engage with an artwork cognitively and their aesthetic valuation of it is likely to depend on a shifting balance between prior expectations and novelty.

#### Artworks and Artistic Practice as Novel Tools to Elucidate the Social Brain Connectome

Artworks—as unique examples of socially relevant sensory objects with contextually dependent salience—have a high potency to act as touchstones for engaging the social brain connectome. Moreover, they are able to distill mental feeling states that are disengaged from any specific human “carrier”—and indeed, often from the artist’s own mental state while creating the artwork—yet remain (like many inter-personal exchanges) ambiguous and dynamic, requiring an active “theory of mind” to decipher them. As experimental stimuli, artworks therefore both engage the social brain, and allow social brain responses to be deconstructed more or less precisely over different processing dimensions (for example, the trajectory of eye movements scanning the perceptual structure; the neural dynamics of semantic and emotional feature encoding). As social artifacts, artworks might also serve as an informative test case in the longstanding debate in social neuroscience concerning the respective roles of theory-based, imitation-based, and narrative-based routes to social understanding (Alcalá-López et al., 2019). We therefore propose that artworks may be candidate “probes” of social brain operation with relevance extending widely beyond art.

The dominant discourse in academic circles is verbal, and this is one important factor that has biased research paradigms and knowledge frameworks toward the domain of verbal semantics. This bias is also apparent in the functional profiles of the social brain connectome and was addressed by the authors as a methodological shortcoming reflecting the available neuroimaging research literature (Alcalá-López et al., 2017). To correct this imbalance, more research is urgently needed to elucidate non-verbal modes and dimensions of complex social thought processes and knowledge exchange. Art communicates with us in many such dimensions, encompassing sensory, emotional, dynamic, social, and conceptual aspects of how we understand ourselves, other people, and the world we live in Arnheim (1969), Goodman (1978), and Bruner (1986).

We propose that artistic practice could be used as a method to generate novel insights into multi-modal aspects of the social and the artistic brain connectome. This approach will require close collaborations between practicing artists, cultural partners, and cognitive neuroscientists, in order that non-verbal embodied, social, and material perspectives are foregrounded in the development and dissemination of novel research paradigms. While fruitful collaborations between artists and neuroscientists are not a novel phenomenon, in practice the theoretical framework, research questions and methodology of such projects have tended to be devised and led primarily by scientists. Outputs are likewise mostly tailored to traditional platforms of academic knowledge exchange, and predominantly expressed in verbal language, which limits the scope of the meaningful contributions that artists and creative methods can make to further our understanding of the multi-modal dynamics of art engagement and complex social thought processes.

### Clinical Resonance of the Artistic Brain Connectome

The strong overlap between the neural dynamics of art processing and social cognition in the artistic brain connectome raises clinical implications that may suggest practical diagnostic and therapeutic applications. We outline some of these within the broad category of aging and dementia syndromes, in this section and in Table 2.

**TABLE 2:** Some diagnostic and therapeutic considerations in clinical application of artworks for healthy aging and dementia.

Target group	Relevant artistic brain connectome mechanisms*	Preserved capacities^†^	Putative deficits	Candidate diagnostic markers	Candidate therapeutic strategies—outcomes
Healthy older people	Altered construction network connectivity	Theory- and narrative-based mental state decoding	Embodied imitation and perspective taking	Reduced social connectedness, loneliness, mood (in relation to healthy older socio-cultural peers)	Enhanced self-expression, social connectedness, mindfulness, resilience, prevention of dementia
Alzheimer’s disease	Altered animation/construction (default-mode) network connectivity	Narrative-based mental state decoding, novelty coding, emotional reactivity	Integration with autobiographical record, perspective taking, visual scene parsing	Impaired processing of visual gestalt (e.g., symbolic value), reduced self-referential descriptions despite normative emotional responses	Sharing of feelings with caregivers and practitioners, enhanced self-expression, and social connectedness
Parkinson’s disease dementia	Altered perception/interaction (visual, dorsal attention) network connectivity	Mental state decoding	Visual scene parsing, emotional reactivity	Impaired parsing of visual features/processing of visual gestalt	Sharing of feelings with caregivers and practitioners, self-expression, and social connectedness
Behavioral variant frontotemporal dementia^¶^	Altered Animation/Interaction/Construction Network connectivity	Creativity, perceptual analysis	Socio-emotional conceptual knowledge, salience coding, emotional reactivity	Socially uncalibrated judgments and emotional responses, reduced autonomic reactivity	Scaffolding of pro-social behavior, while creating space for expression of idiosyncratic and creative impulses in social context
Semantic dementia^¶^	Altered animation/construction network connectivity	Creativity, perceptual analysis	Socio-emotional conceptual knowledge, self-concept, emotional reactivity	Socially uncalibrated judgments and emotional responses, altered autonomic reactivity (e.g., enhanced valuation of particular colors)	Scaffolding of pro-social behavior, while creating space for expression of idiosyncratic and creative impulses in social context

In this table shows putative artistic brain connectome changes and art-based diagnostic and therapeutic applications in healthy aging and some major dementia syndromes (see text). Our intention here is to indicate how neuroscientific progress in elucidating the artistic brain connectome and links to social cognition might be used to tailor art-based interventions in a relevant clinical context. *Proposed leading alteration; ^†^may vary between individuals from relatively spared to positively enhanced; ^¶^these two syndromes show substantial clinical and neuroanatomical overlap.

#### Healthy Aging

Healthy aging is associated with a complex profile of cognitive change (Spreng and Turner, 2019) characterized by gains in some domains and losses in others. Based on a review of the neuroimaging literature on changes in functional connectivity patterns in healthy aging, the default-executive coupling hypothesis of aging emphasizes greater cognitive flexibility in younger adulthood and more crystallized understanding of oneself and the wider world in older adulthood (Spreng and Turner, 2019). In line with this hypothesis, at least some aspects of theory of mind processing may actually improve in later life (Happé et al., 1998). However, data on mentalizing ability in older adults are conflicting (Castelli et al., 2010; Alcalá-López et al., 2019; Martin et al., 2021), perhaps reflecting the nature of the experimental task and the age range (younger-old/oldest-old) assessed. Older adults may be more reliant on theory-based decoding of others’ mental states (an operation mediated chiefly by dmPFC) vs. embodied simulation of another’s perspective (mediated chiefly by TPJ), though functional alterations affecting both components of the Construction Network have been reported (Castelli et al., 2010; Alcalá-López et al., 2019; Martin et al., 2021). With respect to one cognitive process of central importance both for art processing and social cognition more broadly—theory of mind—these considerations may suggest that artworks need not be less potent in engaging social brain networks in neurologically healthy older people than in younger adults. In forming a theory of mind about art, older adults might be able to exploit “narratives” codified in artworks with which they share a sociocultural milieu (Alcalá-López et al., 2019), as well as a propensity to use visual structure to achieve social understanding (Martin et al., 2021).

There is clear therapeutic potential here. For example, artworks and the production of art may harness retained social cognitive capacities in the healthy elderly, encouraging reflection on one’s own life experiences and inter-personal connections and fostering resilience in coping with the social isolation, loneliness and disenfranchisement that are significant issues for many older people in the industrialized world, particularly where there is concomitant depression and anxiety. Following the formulation we propose here, art is a primary means to engage the social brain, thereby aligning it with other strategies for enriching sensory experience, mindfulness and social connectedness that promote healthy cognitive aging and protect against development of dementia (Fabrigoule et al., 1995; Sommerlad et al., 2019). Further, our formulation leads to specific hypotheses concerning how art-based interventions should be designed, targeted, and evaluated (Table 2), building on recent evidence that social dislocation has a neural signature in brain structures—such as DMN—that are integral to the artistic brain connectome (Spreng et al., 2020). Although much of the literature on social prescribing (Fancourt and Finn, 2019; Fancourt et al., 2020) has emphasized clients working with art materials to produce artworks, the social brain hypothesis suggests that the therapeutic scope of art is potentially much broader. Programmed exposure to artworks and eliciting responses to them using approaches such as Visual Thinking Strategies (VTS), a widely used arts-based facilitated conversation method (Housen, 1987, 1999, 2002; Miller et al., 2013; Yenawine, 2013), could open up new directions for developing neuroscientifically informed art therapies.

#### Brain Disorders—The Paradigm of Dementia

Setting art in the social brain is particularly relevant to the diagnosis and potential therapy of dementia, not only because of the scale of human suffering caused by dementia but because the neural network basis of neurodegenerative diseases is increasingly well defined. These disorders cause profound disruption of social brain networks (Greicius et al., 2004; Buckner et al., 2008; Seeley et al., 2009; Galvin et al., 2011; Hafkemeijer et al., 2012; Sheline and Raichle, 2013). This neural network disruption is likely to impact both the affected individual’s sense of self and their interactions with other people, particularly due to impaired theory of mind processing. Furthermore, functional connectivity changes in the earliest pre-symptomatic stages of dementia predict subsequent gray matter atrophy (i.e., irreversible neural damage) that will develop over time (Mandelli et al., 2016). However, dementia is not an amorphous entity, and in particular, different dementia syndromes could have different consequences for the person’s response to art, independently of interpersonal variations in art experiences and interests. Failure to recognize this and/or to design hypothesis-led interventions may account in part for the equivocal benefit identified in previous reviews of art therapy in dementia (Deshmukh et al., 2018). Theory of mind abilities, for example, are affected differently in different diseases and at different disease stages, and according to whether others’ mental cognitive states or feeling states are being interpreted (Bora et al., 2015; Dodich et al., 2016), leading to disease-specific hypotheses about how artworks might be optimally targeted for diagnostic and therapeutic ends (Table 2). Here we illustrate this with two contrasting dementia syndromes—behavioral variant frontotemporal dementia and Alzheimer’s disease.

The behavioral variant of frontotemporal dementia profoundly diminishes the ability to understand and appropriately respond to social cues early in the course of the illness (Sivasathiaseelan et al., 2019). This severe social cognitive phenotype reflects intense and extensive involvement of the anterior hubs of the Animation, Interaction and Construction Networks with socially uncalibrated salience decoding, as well as impaired social and emotional conceptual knowledge. In the context of the artistic brain connectome, this syndrome is predicted to lead to strongly incongruent emotional responses to artworks or idiosyncratic interpretations that appear strongly disconnected from the depicted subject or emotional tone of the artwork. On the other hand, the liberation of non-normative “knight’s move” neural processing mechanisms may at least in part account for the apparently paradoxical flowering of artistic creativity that is a striking feature of the illness in some people with frontotemporal dementia (Erkkinen et al., 2018).

Alzheimer’s disease on the other hand typically predominantly affects medial temporal hubs of the Animation Network (especially the hippocampus) and posterior and temporal hubs of the Construction Network. This profile of network disruption causes people to struggle with recalling the details of autobiographical experiences, as well as with perceptual decoding of complex visual scenes and assuming others’ viewpoints, whereas emotional awareness and prosocial concepts and impulses often remain relatively intact well into the disease process (Bosch-Domènech et al., 2010; Dodich et al., 2016). Moreover, people with Alzheimer’s disease (in contrast to frontotemporal dementia) have retained capacity to process and react appropriately to novel or incongruous features in complex stimuli such as music (Benhamou et al., 2021); this cognitive operation is also likely to be highly relevant to engaging with artworks. In the context of the artistic brain connectome, this dementia profile could be characterized by difficulty with decoding perceptual relationships and/or symbolic meanings from art and/or in finding the right words to describe one’s thoughts and perceptions, despite retained appreciation of the emotional resonance of artworks.

These judiciously chosen examples suggest how the diagnostic application of artworks in the memory clinic might probe processes—such as theory of mind and emotional reactivity—that differentiate early dementia from healthy aging and between dementia syndromes. We propose that arts-based methods have broader potential for the clinical evaluation of social cognition as well as novel therapeutic strategies that extend widely beyond the memory clinic. However, much more research is needed to contextualize arts-based methods in the social and artistic brain connectome across the lifespan, and the gradations that occur in diverse states of mental and neurological brain health.

## Conclusion and Future Directions

We argue, based on the evidence we have presented, that art is inherently a social construct and for this reason, art engagement recruits the same brain networks as complex social behavior. Whereas Kantian perspectives on neuroaesthetics focus heavily on supposed universal concepts of beauty which are disconnected from personal desires, we propose instead that the meaning and experience of an artwork is always created in a social context. Art allows us to reflect on the world around us, but the effects of a particular artwork cannot be assumed to be universal: this will be highly dependent on the viewer’s personal knowledge and experiences and their social and cultural environment.

As a social object, art both reflects and promotes our engagement with the social world at large, and thus our mental wellbeing. This suggests one possible answer to the question, “What is art *for*?” It may help us understand and better negotiate the often surprising social environment we must inhabit as social creatures. Further, art potentially constitutes a novel and powerful tool to understand, diagnose and treat disorders of the socio-emotional brain. In proposing this neuroscientific framework of art perception and production that is grounded in the social brain, we aim to lay the foundation of a functional brain model that can guide future research in this field as well as its practical application in societal and clinical contexts. We hope that our framework will be of use in contemporary art practice and education as well as neuroscientific research and ultimately clinical practice.

The approach we have outlined has several limitations that should direct future work. This is particularly pertinent to the mapping of art and visuospatial creativity onto the social brain connectome. We have aligned our “artistic brain connectome” with the 36 core social brain areas as defined by Alcalá-López et al. (2017), which necessarily excludes a number of other brain areas that have been reported in the neuroaesthetics literature. For instance, the right dorsolateral prefrontal cortex (dlPFC) has been found to be engaged when watching pictures of unnaturally colored objects (Zeki and Marini, 1998). It has also been shown to play an important role in critical self-evaluation of verbal creative thought or expressions (Beaty et al., 2018), while in visuospatial creative thought generation bilateral activation of the dorsolateral prefrontal cortex has been reported (Ellamil et al., 2012; De Pisapia et al., 2016). In the social brain connectome, functional connections between the dlPFC and areas in the Intermediate (Interaction) Network were reported, but the dlPFC itself was not considered part of the core Social Brain Atlas by Alcalá-López et al. (2017). Similar considerations may well apply to other brain regions. Further correlative work, combining qualitative with quantitative methods, is required to refine and expand on the “artistic brain connectome” we have presented here.

A critical test of the “artistic brain connectome” paradigm will be to establish how the connectome is engaged when producing and experiencing artworks, using both lab-based functional neuroimaging techniques and wearable technology that can capture physical responses and cerebral processing directly and in real-time. In recent years, a start has been made with mapping *in situ* art experiences to functional brain networks (Cruz-Garza et al., 2017; Herrera-Arcos et al., 2017), but important factors that need further attention are i) the quality of data acquisition and ii) the impact of the measurement instruments on the art experiences.

Closer collaborations between artists, (arts) educators and social cognitive neuroscientists are needed to investigate the materiality and experiential dimensions of art production and engagement and unpack big concepts like “aesthetic response” and “creativity”—to get at their neural building blocks so we can better understand them. But alongside the deconstruction and reductionism that neuroscience seeks, this process also needs reintegration, to capture our experience of art in the world at large. VTS, for example, offers a non-directive method to engage audiences with art from their personal perspective in a social setting and is ideally placed to further elucidate how art engages the social brain.

Finally, and building on the above, we believe that our framework could also have value in clinical contexts, which we demonstrated with the example of aging and dementia. Neuroscientifically informed art-based interventions could build directly on recent progress in elucidating the social brain connectome in neurodegenerative diseases (Greicius et al., 2004; Buckner et al., 2008; Seeley et al., 2009; Galvin et al., 2011; Hafkemeijer et al., 2012; Sheline and Raichle, 2013), and emerging evidence that the sensory environment influences neural plasticity and evolution of neurodegenerative pathology (Mandelli et al., 2016). The nature and degree of engagement with artworks as diagnostic and therapeutic targets will be influenced by personal interests, experiences, and abilities, but are also likely to differ between dementia syndromes (Table 2). Moreover, art could be a seminal means for promoting social engagement and protecting against development of dementia (Fabrigoule et al., 1995; Sommerlad et al., 2019). Nurturing social brain networks through art engagement might promote psychological well-being and cognitive functioning in healthy aging, as well as mitigating the effects of dementia. Analogous arguments could be made for other developmental, psychiatric, and neurological brain disorders, in which the capacity of art to engage the social brain could create far-reaching therapeutic opportunities.

## Author Contributions

JL conceived of the presented idea, connected art processes to the social brain connectome, conducted the literature review, conceptualized the practical applications of the “social brain connectome,” and created the visual representations of the social brain atlas presented in this work. The social brain connectome was published by the research group led by DB, who also verified the references to the social brain connectome in this work. JW, JB, and SC supervised the conceptualization, the contextualization and formal structure of this work. All authors discussed the ideas put forward in this work and contributed to drafting the manuscript and approved the final version.

## Conflict of Interest

JL was the founder of the Thinking Eye, a social enterprise which translates novel insights from research into relationships between visual art processes and the social brain into services that aim to support psychological wellbeing and optimal cognitive functioning. The remaining authors declare that the research was conducted in the absence of any commercial or financial relationships that could be construed as a potential conflict of interest.

## Publisher’s Note

All claims expressed in this article are solely those of the authors and do not necessarily represent those of their affiliated organizations, or those of the publisher, the editors and the reviewers. Any product that may be evaluated in this article, or claim that may be made by its manufacturer, is not guaranteed or endorsed by the publisher.
